# Metabolic Screening and In Silico Investigation of *Eucalyptus globulus* Essential Oil Bioactives Against DENV‐2 Envelope Protein

**DOI:** 10.1002/open.70307

**Published:** 2026-10-25

**Authors:** Arslan Afzal, Ilyas Ahmad, Mahadullah, Saliha Qamar, Tayyaba Yousaf, Faiz Ullah, Dilawar Khan, Mohammad Hamid Hamdard, Zia ur Rehman Mashwani

**Affiliations:** ^1^ Faculty of Agricultural Engineering & Technology PMAS Arid Agriculture University Rawalpindi Pakistan; ^2^ Department of Botany Arid Agriculture University Rawalpindi Pakistan; ^3^ Department of Entomology PMAS‐Arid Agriculture University Rawalpindi Pakistan; ^4^ Botany Department Faculty of Biology Kabul University Kabul Afghanistan

**Keywords:** bioactive, dengue, essential oil, *Eucalyptus globulus*, network pharmacology

## Abstract

The escalating prevalence of viral infectious diseases poses a significant threat to global public health. Among these, dengue virus (DENV) represents one of the most widespread arboviral infections, impacting nearly 400 million individuals annually, with severe clinical manifestations occurring in approximately 1% of cases, as reported by the World Health Organization (WHO). Extensive research across both academic and industrial sectors has focused on various aspects of DENV, including its molecular virology, epidemiological patterns, transmission pathways, and therapeutic targets. The development of the tetravalent CYD‐TDV (Dengvaxia) vaccine marked a critical advancement in dengue prevention. Nonetheless, emerging evidence has highlighted several limitations associated with current vaccination strategies, prompting a growing emphasis on the discovery and development of antiviral agents as alternative or complementary therapeutic options for effective dengue management. A total of 14 major bioactive compounds were found to be highly correlated with Dengue, yielding 70 common targets between dengue and bioactive compounds. Protein–protein interaction and network pathway analysis by KEGG and Metascape analysis show higher degree of correlation for DENV‐2 targets. Molecular docking analysis shows that Globulol and Ecalyptol have higher binding affinity with two receptors, 1oke and 1tg8 f DENV‐2, with binding energies of −8.3 and −7.6. Toxicity and drug‐likeness analyses confirmed the safety of the metabolites for drug development. These findings suggest that *Eucalyptus globulus* essential oil may have potential in the treatment of dengue fever. However, further in vivo studies using animal models are required to validate its therapeutic efficacy.

## Graphical abstract

Network pharmacology and molecular docking identify bioactive compounds from *Eucalyptus* *globulus* essential oil with potential activity against dengue virus, highlighting 70 shared targets and DENV‐2‐associated pathways. Globulol and eucalyptol show strong binding to DENV‐2 receptors, with favorable drug‐likeness and toxicity profiles, supporting their potential as candidates for further anti‐dengue investigation.

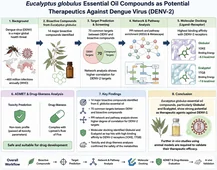

## 1 Introduction

In humans, the re‐emergence of various zoonotic diseases through different species is a well‐documented phenomenon. In the 21st century, several zoonotic outbreaks have been reported. Factors such as climate change, animal husbandry practices, and interactions between wildlife and humans have contributed to these disease outbreaks. These zoonotic infections not only cause several diseases but also result in a number of mortalities from infections caused by Ebola and Marburg viruses [1]. The outbreak of infectious diseases is linked to fear, anxiety, and uncertainty, often accompanied by the spread of unverified information [2]. The Dengue virus (DENV) is a member of the Flaviviridae family, which comprises over 70 significant human pathogens [3]. These viruses predominantly impact tropical and subtropical regions, which are home to approximately 3.9 billion people. Dengue virus is primarily spread by *Aedus aegypti*, with four serotypes: DENV‐1, DENV‐2, DENV‐3, and DENV‐4, with all serotypes having the ability to cause human infection [4]. Epidemiological studies show that Dengue virus (DENV) infection affects nearly two‐fifths of the global population, with an estimated 390 million infections occurring each year. Of these, around 500,000 cases require hospitalization and approximately 20,000 result in death. The virus is primarily prevalent in regions such as Southeast Asia, the Eastern Mediterranean, Africa, the Western Pacific, and South America [5].

The Dengue virus (DENV) possesses a single‐stranded, positive‐sense RNA genome approximately 11 kilobases (kb) in length [6]. This genome encodes a single, continuous polyprotein, which is subsequently processed by virus‐encoded proteases into three structural proteins—capsid (C), envelope (E), and membrane (M)—as well as seven non‐structural proteins: NS1, NS2A, NS2B, NS3, NS4A, NS4B, and NS5. In addition to these coding regions, the genome also contains non‐coding regions (NTRs), particularly at the 3^′^ end, which play important roles in viral replication and regulation [7]. Since the year 2000, there has been a significant increase in the incidence of dengue fever (DF), accompanied by the expansion of cases into new countries and a growing risk of transmission from urban to rural areas—threatening nearly half of the global population [8]. The geographic reach of DENV infections has now extended to 129 countries, including 36 that were previously considered dengue‐free by the World Health Organization (WHO) and the U.S. Centers for Disease Control and Prevention (CDC) [9]. It is estimated that around 390 million DENV infections occur annually, of which 67–136 million present clinical symptoms, with thousands progressing to severe disease or death—primarily in tropical and subtropical regions. This upward trend is attributed to factors such as globalization, population growth and urbanization, climate and environmental changes, inadequate sanitation, ineffective mosquito control measures, and improved surveillance and reporting of DENV cases [8].

Although a large number of people are currently affected, there are still no approved treatments or effective vaccines available to combat dengue fever. Thus, it is crucial to urgently advance research and development efforts to create effective antiviral medications to address this deadly viral infection.

Plants always provide a solution to almost every human disease in one way or the other; same is the case for these life‐threatening viral diseases, for which plants are the most potent antiviral agents. *Euclyptus,* belonging to the family Myrtaceae, is native to Tasmania and Australia, including 900 species mostly located in subtropical climatic zones [10]. *Eucalyptus globulus* is a well‐known plant of the genera, as its essential oil is globally utilized in pharmaceuticals, perfumes, food products, and cosmetics. This oil has demonstrated antiviral, antibacterial, anti‐inflammatory, and antioxidant properties [11]. According to research, it could have some antiviral properties against some viruses. For instance, research indicates that eucalyptus oil may have antiviral properties against respiratory viruses, including the herpes simplex virus and influenza, albeit the exact virus and oil concentration used will determine how effective the oil is.

In the management of complicated disorders, systems biology, compound‐gene set enrichment analysis, polypharmacology, and network pharmacology have been employed recently to comprehend the biological activity of phytoconstituents with numerous protein targets and linked pathways [12]. The network pharmacology idea, in contrast to the “one drug, one gene, one pathway” method, concentrates on the “multi‐compounds, multi‐genes, and multi‐pathways” in the treatment of disease and aids in the investigation of the intricate interactions between compounds and targets [13].

Therefore, this study was conducted to elucidate the biochemical characterization of *E. globulus* essential oil through gas chromatography mass spectrometry (GCMS) analysis and to check the potential of its bioactive compounds against receptors of dengue virus.

## 2 Materials and Methods

### 2.1 Sample Collection and Essential Oil Extraction

The PV‐powered solar distillation system utilized in this study comprised photovoltaic (PV) panels, a distillation still, a condenser, a Florentine vessel equipped with a flask, and a control box for temperature monitoring at various points of the system. *Eucalyptus globulus* leaves were collected from Rawalpindi region Pakistan; on‐site botanical identification was done by the expert botanists of Department of Botany, Arid Agriculture University, Rawalpindi, Pakistan, with the help of botanical description and distinguishing characters. The experiments were conducted using 10 kg fresh leaf batches of *Eucalyptus globulus*. The steam flow rate was calibrated. A total of 20–30 liters of water were utilized in the distillation chamber, and the moisture content of the plant material was recorded prior to distillation. The distillation process began with steam generation, which was then followed by the evaporation of essential oils from the plant material through diffusion. The steam and essential oil volatile essence were moved into the condensation chamber and then into the Florentine vessel, where the oil and water were separated based on their density differences. The extracted essential oil was carefully collected and refrigerated at 4 °C for preservation. Each distillation experiment was expected to take approximately 2–3 h to complete, with multiple batches processed on a sunny day to optimize the use of solar energy for essential oil extraction. The total concentration of essential oil from the 10 kg batch was 45 ml.

### 2.2 Larvicidal Bioassay

The Larvicidal activity of *Eucalyptus* essential oil was evaluated against fourth‐instar (IV‐Instar) mosquito larvae following the methodology of Wang et al., with slight modifications. Fourth‐instar larvae were selected and maintained under laboratory conditions prior to the experiment. A stock solution of *Eucalyptus essential oil* was prepared using an appropriate emulsifying agent (i.e., Tween 20) to ensure uniform dispersion of the hydrophobic essential oil in water. From the stock solution, five test concentrations (62.5, 125, 250, 500, and 1000 ppm) were prepared by serial dilution with dechlorinated water. For each concentration, 30 IV‐Instar larvae were introduced into separate experimental containers, while each treatment was performed in triplicate. An untreated control group containing larvae maintained in dechlorinated water without essential oil was used as a negative control. Temephos, a commercially established organophosphate larvicide, was used as the positive control to validate the sensitivity of the bioassay and compare the larvicidal efficacy of the Eucalyptus essential oil. The larvae were exposed continuously to the test solutions under controlled laboratory conditions, and larval mortality was recorded at 24, 48, 72, 96, and 120 h after treatment. Larvae showing no movement after gentle probing with a fine brush or glass rod were considered dead. Dead larvae were removed after each observation to avoid interference with subsequent observations, while surviving larvae remained exposed to the respective treatment throughout the experimental period [14]. The percentage mortality at each exposure interval was calculated by using the formula,

Mortality(%) = Total number of larvae exposed/ Number of dead larvae × 100

The concentration and time‐dependent mortality data were subsequently subjected to probit analysis to determine the median lethal concentration (LC_50_) and lethal concentration causing 90% mortality (LC_90_), together with their corresponding 95% confidence intervals, at each exposure period. The experiment was conducted under identical environmental conditions for all treatment and control groups to ensure reproducibility and minimize experimental variation.

### 2.3 Gas Chromatography Mass Spectrometry (GCMS) Analysis of Oil Samples

The extracted essential oil of *E. globulus* was subjected to GCMS analysis at the Centralized Resource Laboratory at the University of Peshawar in Pakistan. Analysis was done using the PerkinElmer Clarus 500 model, which has FID, TCD, FPD, and ECD capabilities. The temperature was maintained at 70 °C, and helium gas was utilized as the carrier gas. The chemicals in the oil sample were identified by running the observed peaks through ChemStation and the NIST11.L compound database. By contrasting the compounds’ retention duration and molecular weight with previously published information in the literature, further compound confirmation was accomplished by GCMS analysis.

### 2.4 Identification of Compounds

*E. globulus* bioactive compounds were identified using the NIST library, m/z values, and by comparing with already available literature to enhance the validity of the compounds. KEGG and PubChem IDs for all compounds were retrieved from databases to make a final list of the compounds for further screening and in silico analysis.

### 2.5 Metabolite and Disease Targets

To evaluate the potential effectiveness of *Eucalyptus globulus* ‍bioactive compounds against dengue fever, the SwissTargetPrediction tool was employed to identify all possible molecular targets of the selected metabolites. Concurrently, disease‐associated genes and targets related to dengue fever were retrieved from reputable databases, including DisGeNET and GeneCards. Duplicate entries were carefully removed to ensure data integrity. The predicted targets of the *E. globulus* metabolites were then cross‐referenced with the dengue‐associated targets to identify common (overlapping) targets, which were subsequently shortlisted for downstream analysis. This integrative approach facilitated a focused exploration of the bioactive potential of *E. globulus* against dengue pathogenesis through shared molecular pathways**.**

### 2.6 GO Enrcihment, Pathway Analysis, and Protien–Protein Interaction (PPI)

All common disease‐metabolite targets were subjected to Gene Ontology (GO) enrichment analysis using the DAVID database to identify the biological processes, molecular functions, and cellular components associated with these targets. Additionally, pathway enrichment analysis was performed to determine the key signaling and metabolic pathways in which these targets are involved in humans. To further understand the functional associations among these targets, a PPI network was constructed and analyzed using the STRING database, providing insights into the potential interactions and regulatory roles of these proteins in disease mechanisms.

### 2.7 Ligand Preparation

All the metabolites were subjected to Pubchem database to retrieve Isomeric smiles, and to get 3D sdf format of all metabolites. Furthermore, the structures in SDF format were converted to PDB format using Biovia Discovery Studio. In AutodockTools‐1.5.7, ligands were prepared by removing water molecules, adding hydrogen bonds, and applying the required charges (Kollman and Gasteiger). The compound structures were then saved in the PDBQT format and utilized for docking.

### 2.8 Receptor Selection, Retrieval, and Validation of Protein Model Quality

Two receptors were selected for docking analysis on the basis of literature available, their importance in disease incidence and survival, and possible drug targets. RCSB PDB database was used for protein structure retrieval, Dengue virus E glycoprotein (PDB ID: 1TG8) and Crystal structure of the dengue 2 virus envelope protein (PDB ID: 1OKE) were retrieved in PDB format. To facilitate a thorough quality analysis and validation of the protein model, we employed the ProSA web server [15], which evaluates the overall quality of the protein structure in each chain (Figure 1).

**FIGURE 1:**
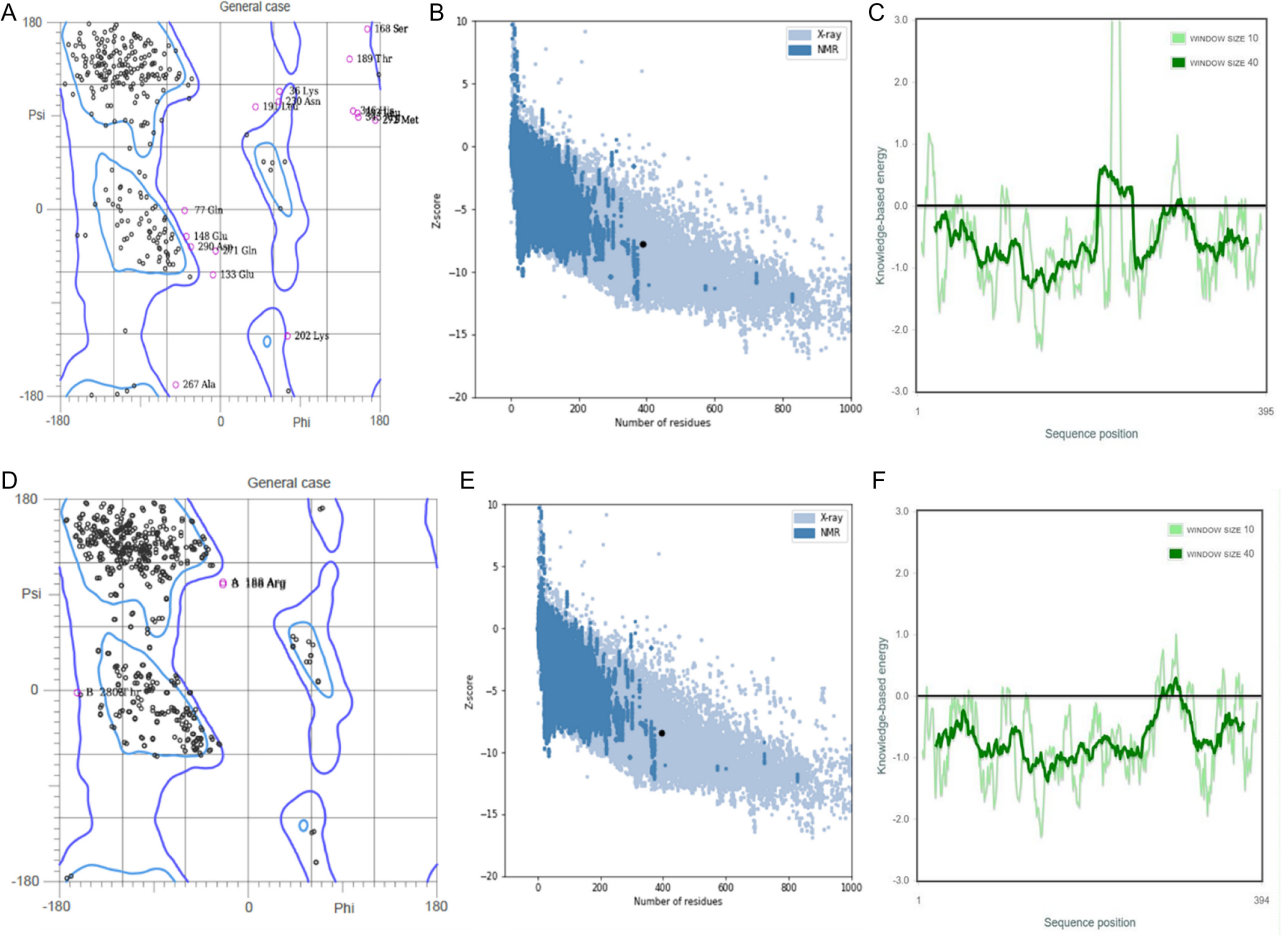
(A–F) Ramachandran graph and Molprobity analysis of selected DENV‐2 receptors.

### 2.9 Purification for Molecular Docking Analysis

The retrieved PDB files of the target receptor molecules were carefully prepared for molecular docking analysis. This preparation involved the removal of all non‐essential components, including water molecules, co‐crystallized ligands, and heteroatoms, to ensure that only the receptor’s active conformation was retained. Following this, polar hydrogens were added, and appropriate partial charges were assigned to the receptor using standard protocols to optimize it for docking studies. The processed receptor structures were then subjected to molecular docking analysis using AutoDock Vina, a widely used and validated tool for predicting ligand‐receptor binding conformations and affinities. This approach ensured the accurate modeling of molecular interactions and binding energies between the ligands and their target sites.

## 3 Results

### 3.1 Larvicidal Activity of *E. globulus* Essential Oil Against *Ades Aegypti*

The larvicidal activity of essential oil against *Ades aegypti* was checked at 4 different time intervals, like 24, 48, 72, and 96 h. After 24 h of exposure, the lowest concentration, 62.5 ppm, showed 33% mortality rate, while the highest concentration, 1000 ppm, induced 73% mortality. After 48 h of exposure, the mortality rate increased significantly across all concentrations; at the lowest concentration, 46%, and at the highest concentration, 80% was observed. At 72 h, the highest concentration showed 80%, and lower concentration showed 60% mortality rates. At 96 h of exposure again, a significant increase in mortality rate was observed, with 66.6% at lower concentration and 86.6% at highest concentration. At an exposure of 120 h, lethality rates were high. At 62.5 ppm, which is the lowest concentration, a 70% mortality rate was observed, and at the highest concentration, about 90% mortality was observed (Figure 2).

**FIGURE 2:**
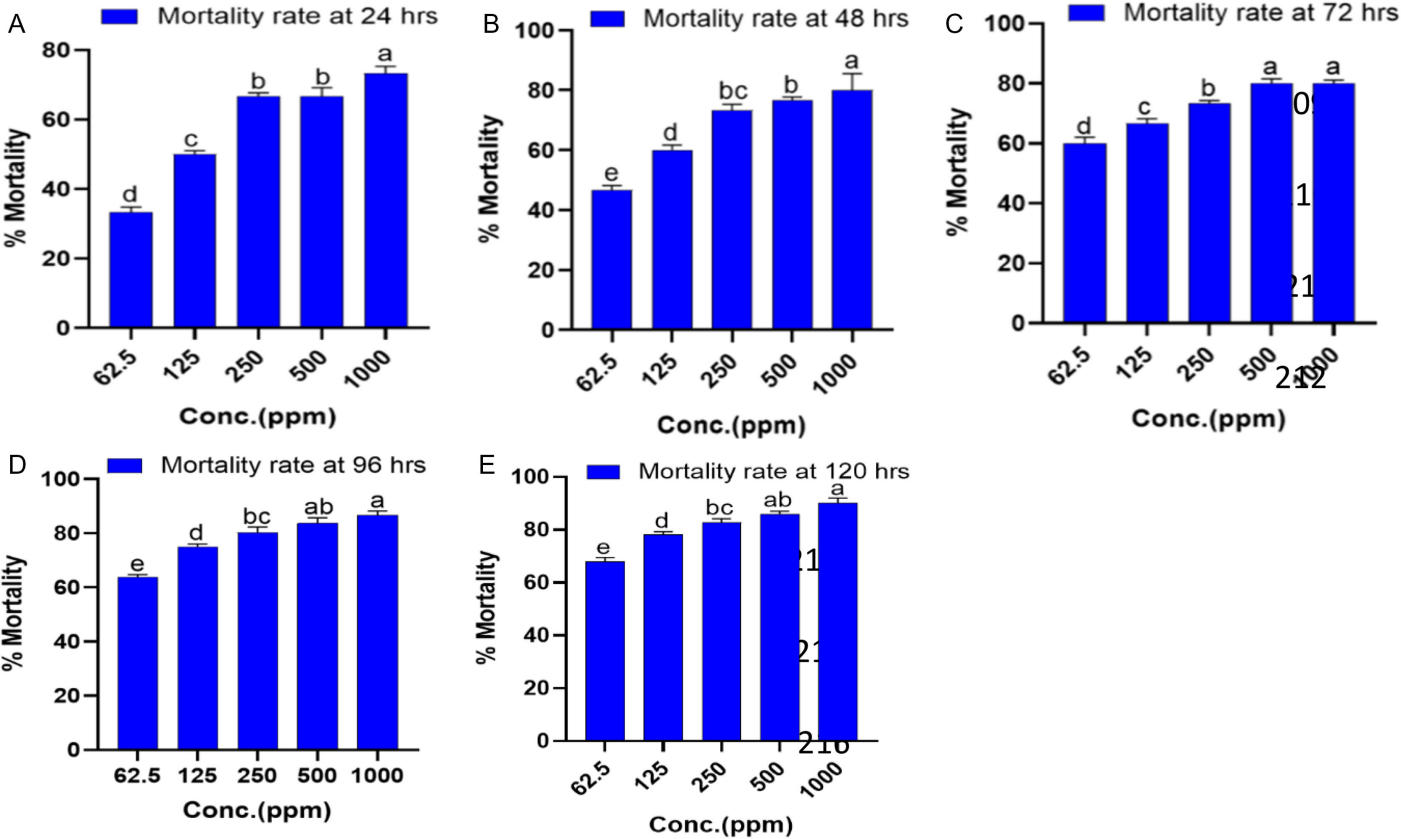
The Larvicidal activity of essential oil against *Ades aegypti* at 4 different time intervals 24, 48, 72, and 96 h.

### 3.2 Phytochemical Profiling of *E. globulus* Essential Oil

From a total of 10 kg of *Eucalyptus globulus* leaf samples, approximately 45 mL of essential oil was extracted through PV‐powered steam distillation. This essential oil was then subjected to gas chromatography–mass spectrometry (GC–MS) analysis to profile its chemical constituents. The analysis revealed the presence of 25 distinct compounds, with D‐Limonene and Eucalyptol (1,8‐cineole) being the most abundant, together constituting over 80% of the total metabolite content. All identified metabolites were found to belong to the terpenoid class, specifically monoterpenoids and sesquiterpenoids, indicating a high richness of these two terpenoid subclasses in the oil. Notably, other significant terpenoids such as (−)‐Globulol and Longifolene were also present in substantial quantities (Table 1).

**TABLE 1:** Major bioactive compounds from GCMS analysis of *E. globulus* essential oil.

No.	Compounds	Formula	Pubchem ID
1	3‐Carene	C_10_H_16_	26049
2	beta.‐Pinene	C_10_H_16_	14896
3	beta.‐Myrcene	C_10_H_16_	31253
4	beta.‐Phellandrene	C_10_H_16_	11142
5	p‐Cymene	C_10_H_14_	7463
6	D‐Limonene	C_10_H_16_	440917
7	Eucalyptol	C_10_H_18_O	2758
8	gamma.‐Terpinene	C_10_H_16_	7461
9	4‐Carene	C_10_H_16_	78249
10	2‐Carene	C_10_H_16_	79044
11	3‐Cyclohexen‐1‐ol, 4‐methyl‐1	C_10_H_18_O	11230
12	Terpinen‐4‐ol	C_10_H_18_O	11230
13	Alpha.‐Terpineol	C_10_H_18_O	17100
14	Decanal	C_10_H_20_O	8175
15	Thymol	C_10_H_14_O	6989
16	3‐Methyl‐4‐isopropylphenol	C_10_H_14_O	18597
17	Bicyclogermacrene	C_15_H_24_	13894537
18	Alloaromadendrene	C_15_H_24_	10899740
19	Epiglobulol	C_15_H_26_O	11858788
20	Longifolene	C_15_H_24_	289151
21	(−)‐Spathulenol	C_15_H_24_O	13854255
22	(−)‐Globulol	C_15_H_26_O	12304985
23	Ledol	C_15_H_26_O	92812
24	1H‐Cycloprop[e]azulen‐4‐ol,	C_15_H_26_O	11996452
25	Di‐n‐octyl phthalate	C_24_H_38_O_4_	8346

Given that many of these compounds, including D‐Limonene, Eucalyptol, (−)‐Globulol, and Longifolene, have been previously reported to possess strong larvicidal properties, the essential oil was further evaluated for its larvicidal activity against dengue vector larvae. This step was taken to validate the potential of *E. ‍globulus* essential oil as a natural, plant‐based larvicidal agent for vector control applications.

### 3.3 Metabolite and Disease Targets

All the metabolites were subjected to Swiss target prediction analysis to check their potential targets. It was found that most of the metabolites found to have targets associated with signaling, immunity, inflammation, and response to external stimuli, which suggest a strong association of these metabolites against Dengue. The GC–MS analysis of *Eucalyptus globulus* essential oil revealed several major terpenoid compounds, including D‐Limonene, Eucalyptol, and Thymol, which are not only abundant but also pharmacologically relevant in the context of dengue fever. Computational target prediction revealed that D‐Limonene is associated with multiple biologically significant target classes, including nuclear receptors, Family A G protein‐coupled receptors (GPCRs), cytochrome P450 enzymes, and various metabolic enzymes. These targets are critically involved in immune modulation, inflammatory pathways, and xenobiotic metabolism—processes often disrupted during dengue virus infection. Similarly, Eucalyptol, another highly abundant metabolite, demonstrated a strong association with targets belonging to the cytochrome P450 enzyme family, as well as key enzymes such as transferases, oxidoreductases, hydrolases, and lyases. These enzymes play a vital role in oxidative stress regulation, detoxification, and metabolic reprogramming, which are known to be implicated in dengue pathophysiology. For Thymol, the predicted targets were enriched in voltage‐gated ion channels, oxidoreductases, and Family A GPCRs, highlighting its potential to interfere with host cellular signaling and ionic balance, which are critical during dengue virus entry and replication. The association of these compounds with such diverse and relevant biological target classes suggests their multi‐targeted potential against dengue fever, not only through direct antiviral action but also by modulating host pathways involved in inflammation, immunity, and viral propagation. These findings provide a molecular basis for the observed larvicidal and therapeutic potential of *E. globulus* essential oil in dengue vector control and possibly in future antiviral drug development.

### 3.4 Metabolite‐Disease Common Targets

All the predicted targets of dengue‐associated genes and *Eucalyptus globulus* metabolites were systematically analyzed to identify overlapping targets. A total of 70 common targets were identified, suggesting a significant degree of interaction between the bioactive compounds and host pathways involved in dengue fever. Notably, a majority of these shared targets were either directly implicated in dengue pathogenesis or indirectly involved in immune regulation, inflammation, and viral replication processes (Figure 3). For instance, CD38 was identified as a key overlapping gene. CD38 encodes a multifunctional enzyme involved in immune cell signaling, cytokine production, and calcium mobilization—processes that are critically dysregulated during dengue virus infection. Moreover, CD38 has been reported as a differentially expressed gene in dengue patients, further underscoring its potential as both a therapeutic target and a biomarker for disease progression [16].

**FIGURE 3:**
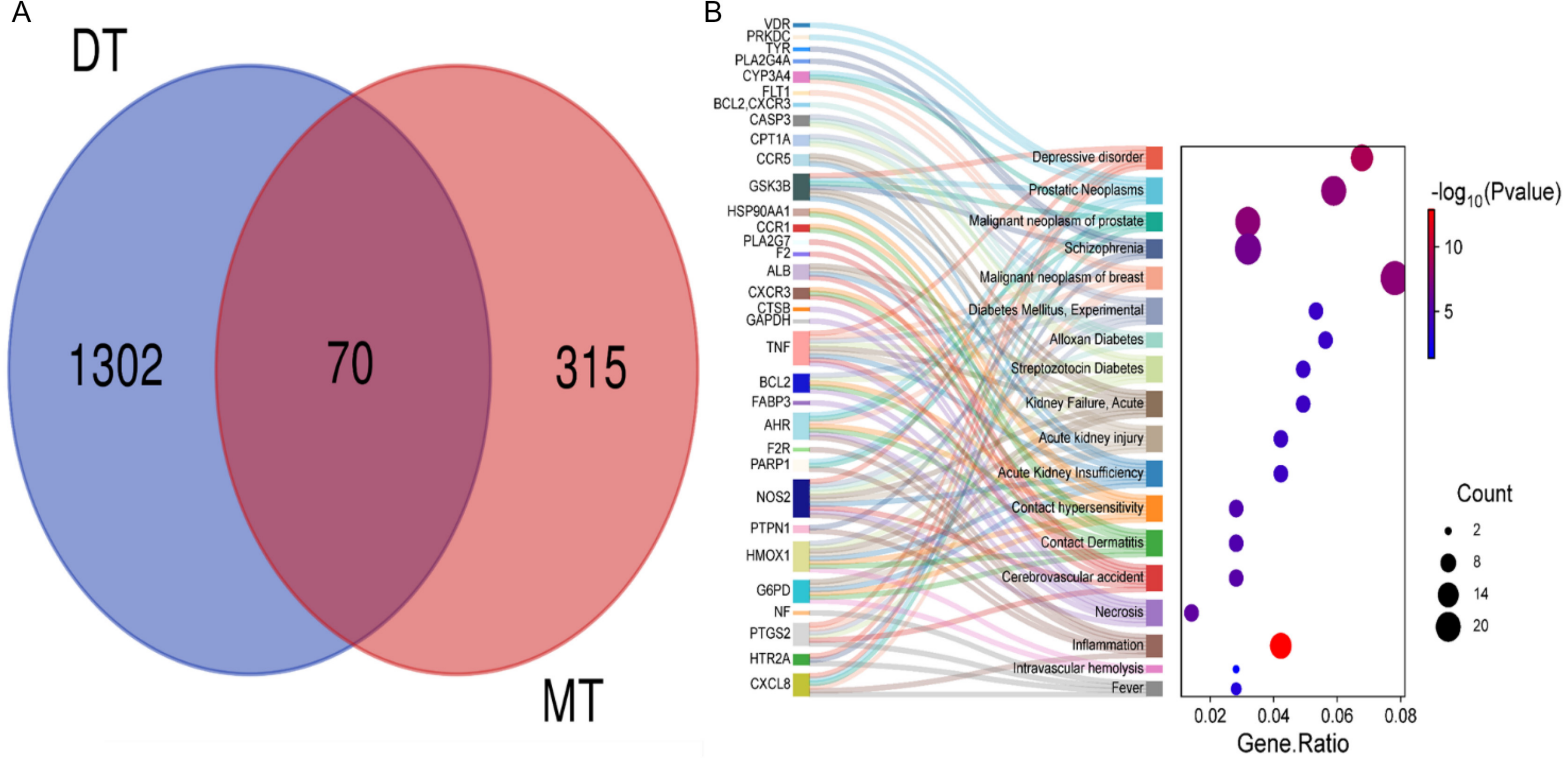
(A) Venn diagram showing common targets between Dengue fever and *E. globulus* essential oil metabolites, (B) network analysis and protein–protein interaction of top proteins and relatedness of DENV‐2 disease targets.

Similarly, other overlapping gene targets, such as MAPK14, were also found to be indirectly associated with dengue fever. MAPK14, a key component of the p38 MAPK signaling pathway, plays a critical role in cellular stress responses, inflammation, and immune regulation. These processes are often activated during viral infections, including dengue. Notably, MAPK14 has also been reported as a differentially expressed gene in dengue‐infected individuals, highlighting its involvement in the host response to viral stress and inflammation. The presence of such common gene targets between *E. globulus* metabolites and dengue‐associated genes supports the hypothesis that these metabolites may exert biological effects through the modulation of dengue‐relevant pathways. The overlapping targets not only reinforce the pharmacological relevance of the identified compounds but also suggest a potential mechanistic correlation between the bioactive components and the molecular landscape of dengue fever. This provides a strong foundation for future experimental validation and potential therapeutic exploration.

### 3.5 Gene Ontology (GO) Analysis

To explore the functional relevance of the common disease‐metabolite targets, GO and pathway enrichment analyses were performed (Figure 4). This helped identify the key biological processes and signaling pathways potentially involved in dengue fever. GO revealed that these common targets were specifically associated with important cellular components, molecular functions, and biological processes. For example, in Molecular functions (MF) class, heterocyclic compound binding and chemokine ligand binding were identified; heterocyclic compound binding showed the lowest and most significant *p*‐value 9.818 × 10^−1^3 and 6.436 x 10^−3^. As many of the dengue‐related enzymes, proteins, and receptors interact with heterocyclic compounds, enrichment of this term suggests that these metabolites, which often contain heterocyclic structures, have the ability to effectively bind to the dengue‐related receptors, potentially interfering with viral replication along with host response mechanisms. Similarly, this involves the interaction with chemokines, which are signaling proteins that regulate immune cell trafficking and inflammation. During dengue infection, cytokine storms and immune dysregulation are major causes of severe symptoms like vascular leakage and hemorrhagic fever. Enrichment of chemokine binding targets (e.g., CXCL8/IL‐8, CCR1, CCR5) suggests that the common genes may play a key role in modulating immune responses. Metabolites targeting these proteins could help in alleviating the hyper‐inflammatory state seen in dengue. As far as the biological processes (BP) enrichment is concerned, major biological processes were viral processes, with a *p‐*value of 2.486 x 10^−3^ which are directly associated with viral pathogenesis, biology, and replication. Further, most of the BP terms were positively correlated with the immune system, like T‐Cell lineage commitment, T‐Cell homeostasis, and leukocyte‐mediated immunity, which shows that these metabolite targets are mainly associated with immune system responses, which are highly affected in dengue fever. Furthermore, cellular components that were highly correlated with these targets were mitochondrial membrane and outer membranes of other organelles.

**FIGURE 4:**
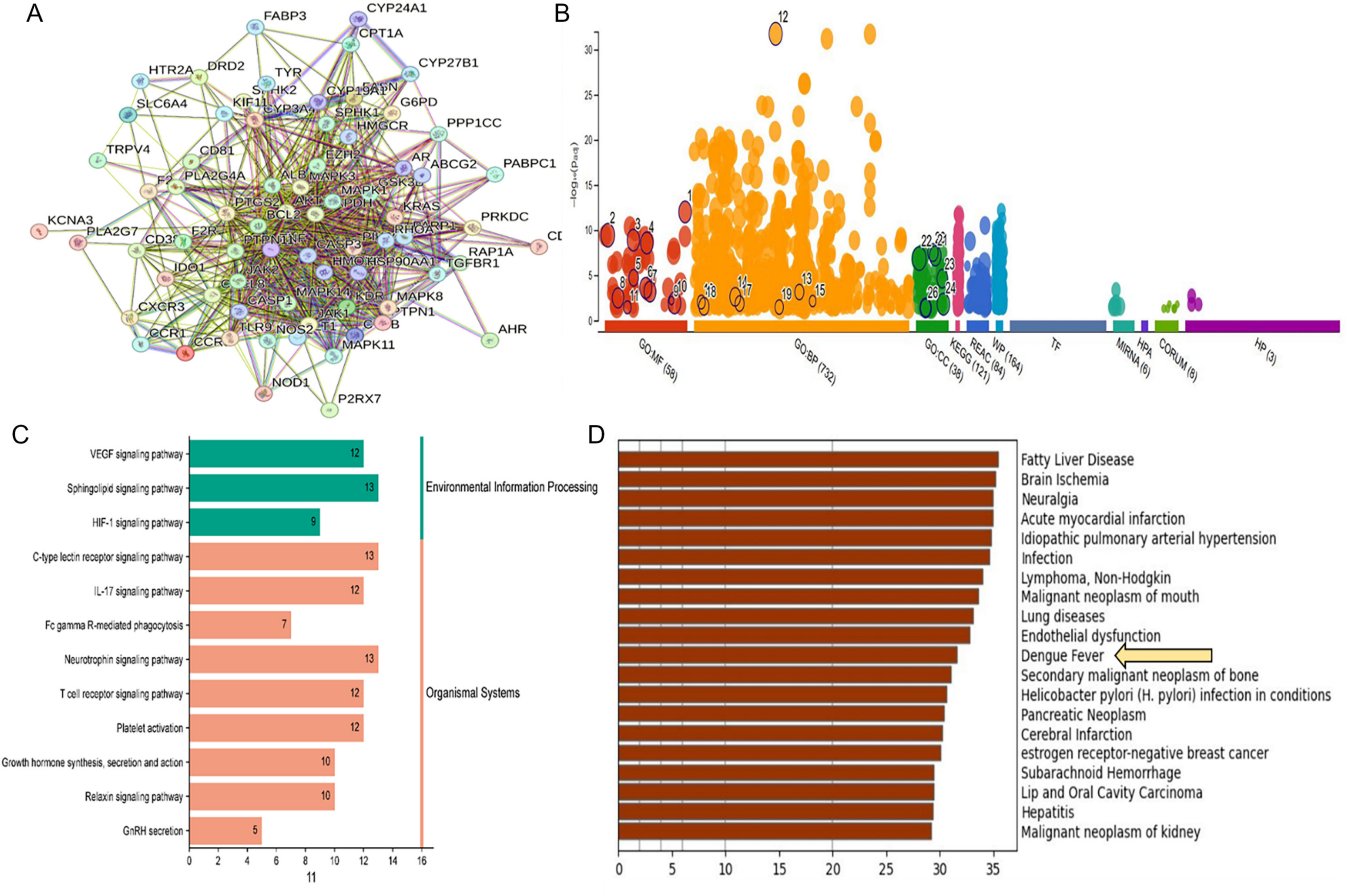
Protein–protein interaction, Gene ontology, KEGG pathway analysis, and Metascape analysis of disease‐metabolite common targets.

### 3.6 Pathway Enrichment Analysis

Similarly, to GO, certain important pathways were also found to be positively correlated with these common targets. For example, all these metabolites were highly enriched in signaling pathways like VEGF signaling pathways, sphingolipid signaling pathway, IL‐17 signaling pathway, T‐cell signaling pathway, and platelet activation. Mean enrichment of these metabolites in the specific pathways suggests a strong interactive ability of *E. ‍globulus* metabolites as a candidate drugs for dengue. This high enrichment in these pathways suggests that these metabolites are not only involved in stress signaling but also activate the immune system to combat. Furthermore, Metascape analysis also showed abundance of all these common targets in important disease. Importantly, 37% of the targets were highly correlated with dengue fever, which shows their involvement not only in the immune system but also directly with dengue fever and its response.

### 3.7 Molecular Docking Analysis

Molecular docking analysis of all selected metabolites was done by using Autodock Vina software. Two dengue receptors, Dengue virus E glycoprotein (PDB ID:1tg8) and dengue 2 virus envelope protein (PDB ID:1oke), were selected for molecular docking analysis. The 1TG8 structure corresponds to the NS3 serine protease of the Dengue virus, a critical enzyme for viral polyprotein processing and replication. Its conserved catalytic triad and well‐characterized active site make it a prime target for antiviral drug discovery. Molecular docking of bioactive metabolites enables the identification of potential protease inhibitors. This strategy facilitates the development of novel, mechanism‐based antiviral agents against dengue. The 1OKE structure represents the envelope (E) protein domain III of the Dengue virus, which plays a pivotal role in viral attachment, host cell recognition, and membrane fusion. This domain is a key target for neutralizing antibodies and small‐molecule inhibitors due to its involvement in viral entry. Molecular docking of bioactive metabolites to 1OKE can reveal potential candidates that disrupt viral‐host interactions. Targeting this domain offers a rational approach for the development of entry‐inhibiting antiviral therapeutics. Molecular docking analysis revealed that Eucalyptol and Globulol exhibited the highest binding affinities toward the 1OKE receptor, representing the envelope protein domain III of the Dengue virus. Eucalyptol showed a binding energy of –7.6 kcal/mol, while Globulol displayed a more favorable binding energy of –8.3 kcal/‍mol. These values reflect strong and thermodynamically stable interactions with the receptor’s active or binding regions. Given the envelope protein’s crucial role in viral attachment and host cell entry, these results suggest that both metabolites may interfere with viral fusion and infectivity, highlighting their potential as entry‐inhibiting antiviral agents. Furthermore, for the 1TG8 receptor, which corresponds to the NS3 serine protease of the Dengue virus, Eucalyptol exhibited the highest binding affinity with a docking score of –7.6 kcal/mol, indicating a strong and stable interaction with the active site of the protease. Limonene followed with a binding energy of –6.8 kcal/mol, also suggesting a favorable interaction, though slightly weaker compared to Eucalyptol. These negative binding energies reflect thermodynamically stable ligand–protein complexes and imply that both metabolites may have the potential to inhibit the enzymatic activity of NS3 by interacting with its catalytic or substrate‐binding regions (Figures ‍5 and 6). Given the critical role of NS3 in viral replication, these findings highlight Eucalyptol and Limonene as promising natural compounds for further investigation as potential antiviral agents against Dengue virus (Table 2).

**FIGURE 5:**
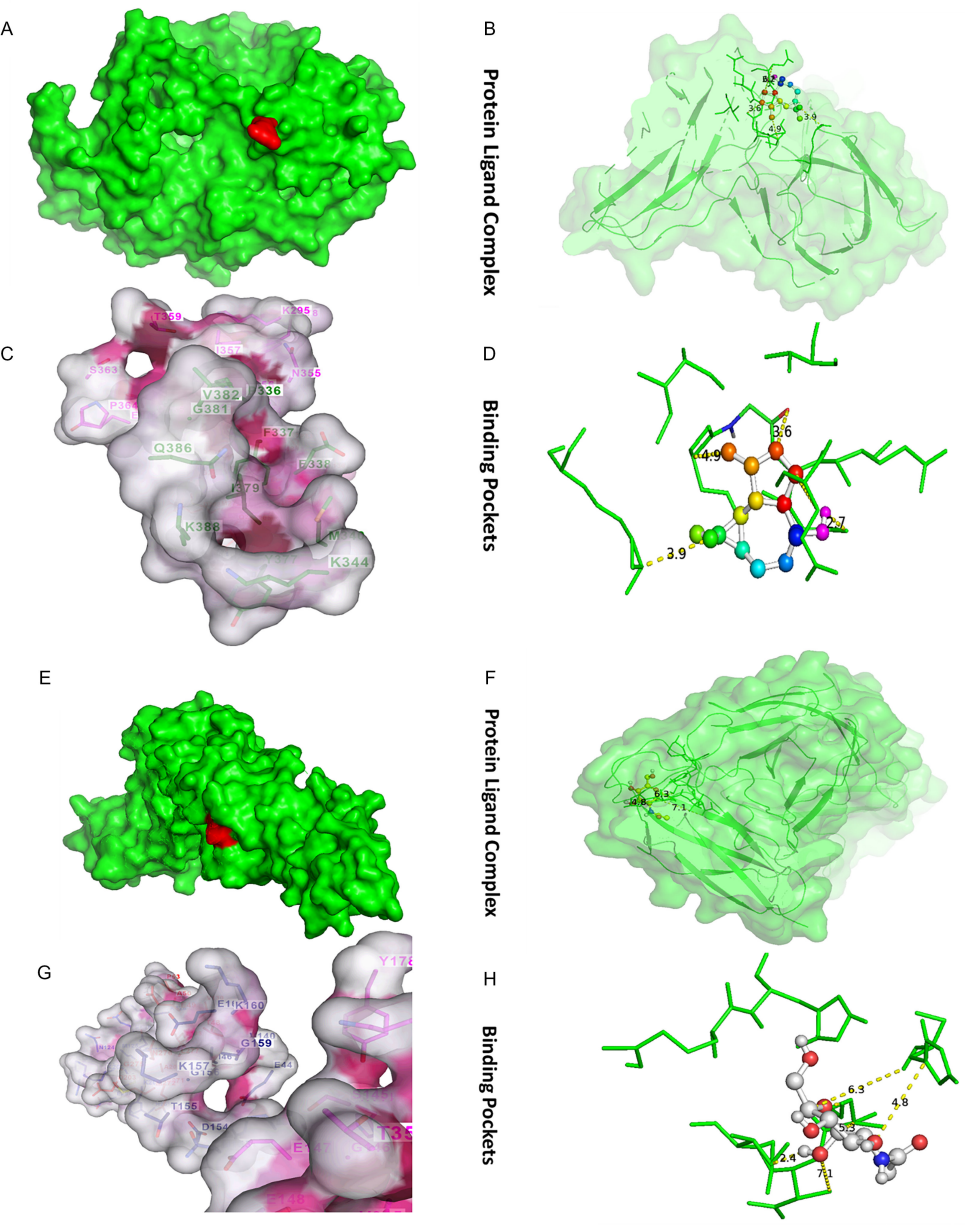
(A–D) Protein ligand complex of Globulol with 1tg8 receptor (A,B) protein ligand complex, (C) binding pockets of receptor for docking and (D) active site of receptor, showing interaction and bonding pattern of Globulol with 1tg8. (E–H) Protein–ligand complex of Eucalyptol with 1tg8 receptor (E,F) protein ligand complex, (G) Binding pockets of receptor for docking, and (H) active site of receptor, showing interaction and bonding pattern of Eucalyptol with 1tg8.

**FIGURE 6:**
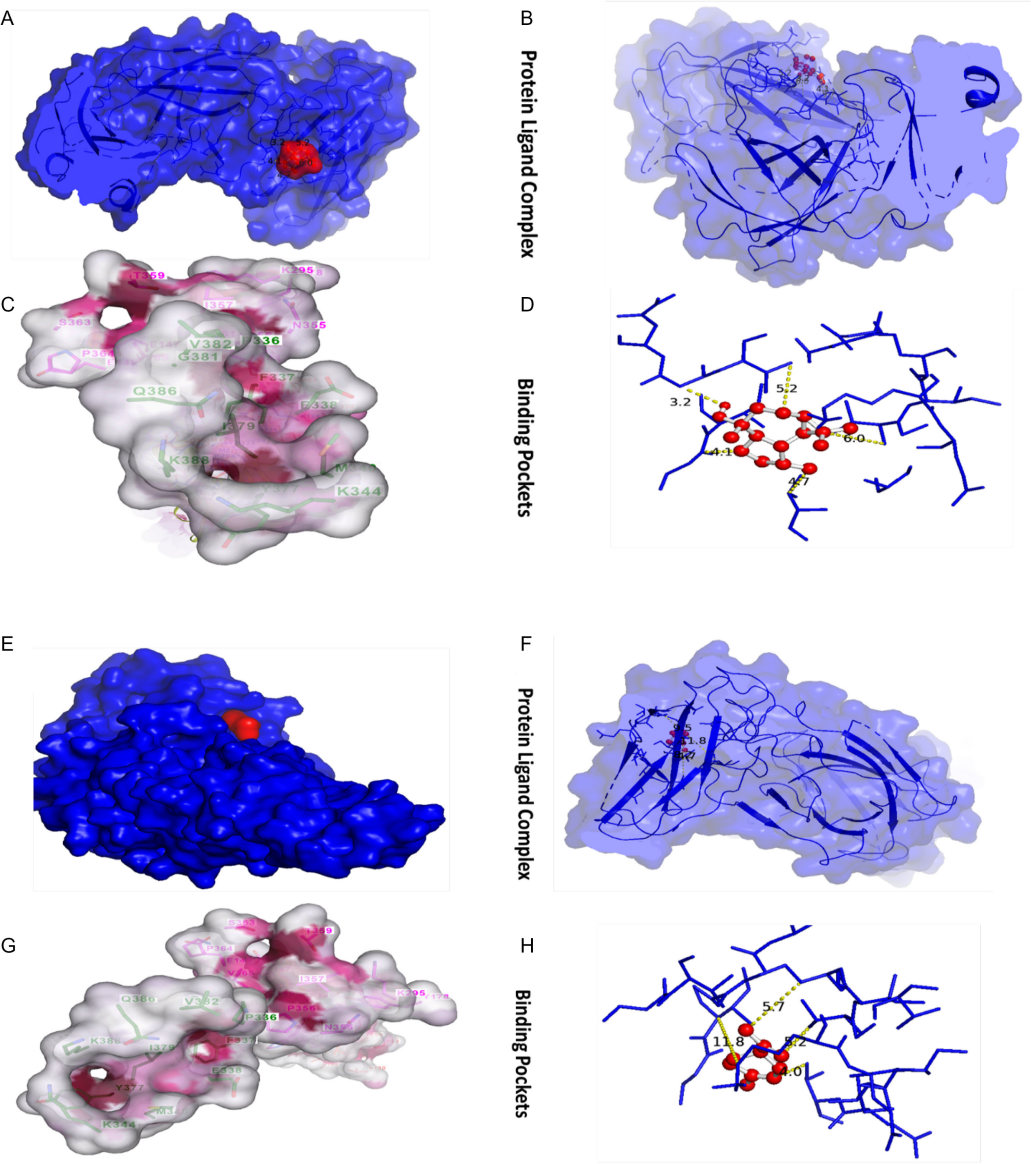
(A–D) Protein ligand complex of Globulol with 1oke receptor, (A,B) protein ligand complex, (C) binding pockets of receptor for docking and (D) active site of receptor, showing interaction and bonding pattern of Globulol with 1oke. (E–H) Protein–ligand complex of Eucalyptol with 1oke receptor, (E,F) protein ligand complex, (G) binding pockets of receptor for docking, and (H) Active site of receptor, showing interaction and bonding pattern of Eucalyptol with 1oke.

**TABLE 2:** Binding affinities of top 2 metabolites against selected DENV‐2 receptors.

Receptor	Metabolite	Binding affinity, kcal/mol
1oke	Eucalyptol	−8.3
	Limonene	−6.8
1tg8	Globulol	−5.9
	Eucalyptol	−7.6

### 3.8 Toxicity Prediction and Lipinski Rule of Five

All the dicked metabolites were further subjected to Protox‐III tool to check their toxicity. It was found that most of the metabolites were found inactive for majority of the targets, Euclyptol and Globulol were found to be completely inactive for hepatotoxicity, neurotoxicity, cardiotoxicity and carcinogenicity, while Thymol and Longifolene were found to be partially active for neurotoxicity. Further, it was also found that Limonene was slightly active for cardiotoxicity while remaining active for all other parameters. The highest LD50 value was noted for Longifolene with 5000 mg/kg, followed by Limonene with 4400 mg/kg and Euclyptol of 2480 mg/kg. This toxicity prediction suggests that these metabolites are safe to be used as drugs as they are mainly non‐toxic to various important targets in humans. Further, all these selected metabolites were subjected to various parameters to determine whether they follow drug‐likeness according to Lipinski rule of five, and it was found that all the selected metabolites fulfilled criteria of Lipinski rule of five and np violation was found. Moreover, which suggests that all these metabolites have drug‐likeness and can further be used for in vivo testing and for commercial drug preparations (Tables 3 and 4).

**TABLE 3:** Toxicity prediction of selected metabolites to check for hepatotoxicity, neurotoxicity cardiotoxicity, and carcinogenicity.

**Hepatoto** **xicity**	Neurotoxicity		Cardiotoxicity		Carcinogenicity	
Metabolite	Predicted LD 50 mg/kg	Toxicity class	Hepatotoxicity	Neurotoxicity	Cardiotoxicity	Carcinogenicity
Eucalyptol	2480	5	Inactive	Inactive	Inactive	Inactive
Limonene	4400	5	Inactive	Inactive	Active	Inactive
Thymol	640	4	Inactive	Partially active	Inactive	Inactive
Longifolene	5000	5	Inactive	Partially active	Inactive	Inactive
Globulol	2000	4	Inactive	Inactive	Inactive	Inactive

**TABLE 4:** Lipinski rule of five for selected metabolites.

Metabolite	**Molecular** **weight (g/mol)**	Log (p)	No of H Bond donors	No of H Bond acceptors	Rule violations
	>500	>5	>5	>10	>2
Eucalyptol	154.25	2.74	0	1	0
Limonene	136.23	3.31	0	0	0
Thymol	150.22	2.82	1	1	0
Longifolene	204.35	4.42	0	0	0
Globulol	222.37	3.47	1	1	0

### 3.9 Swiss ADME Analysis

Swiss ADME analysis of all selected metabolites was checked using Swiss ADME online database. Swiss ADME predicts six major drug‐likeness parameters for the metabolites. Lipophilicity measures the solubility of metabolites in fats; size and molecular weight of the metabolites, which should be less than 500 g/mol; polarity, which explains the total polar surface area of the metabolite; along with insolubility, instauration, and flexibility, which give the number of rotatable bonds. Swiss ADME analysis of all the compounds showed that all metabolites lie in the colored zone of the Swiss ADME diagram, showing their suitability for oral bioavailability (Figures 7 and 8).

**FIGURE 7:**
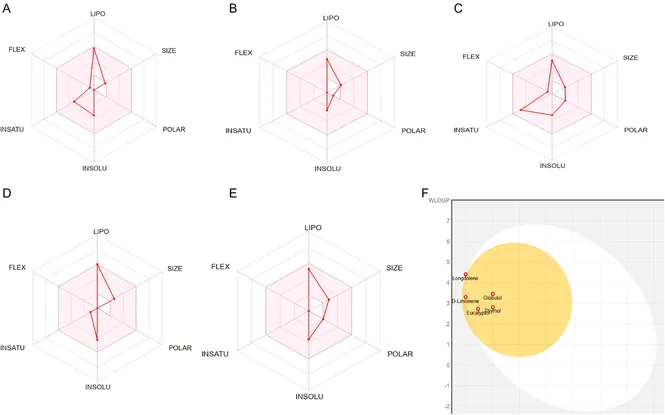
(A–E) Show the different drug‐likeness parameters for the selected metabolites. (F) Boiled‐egg diagram showing the characteristics of the selected metabolites.

**FIGURE 8:**
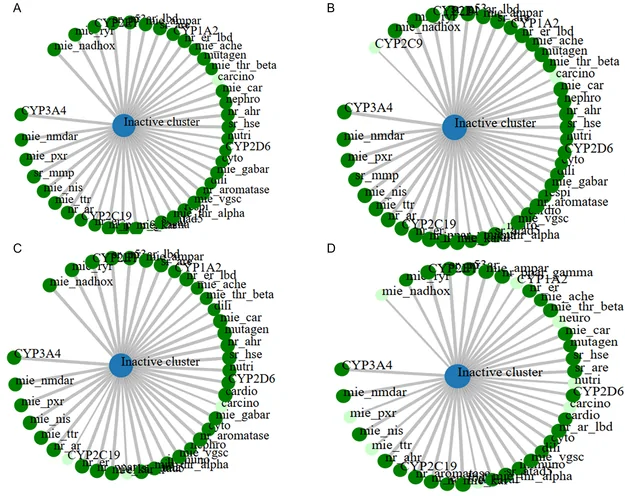
Inactive clusters of all selected bioactive compounds revealed by Swiss ADME analysis.

## 4 Discussion

Medicinal plants serve as a pivotal reservoir for drug discovery and pharmaceutical development, primarily due to their extensive repertoire of phytochemicals spanning multiple structural and functional classes [17]. The chemical diversity inherent in these compounds underpins their ability to exert pleiotropic effects, facilitating multi‐targeted therapeutic mechanisms that contribute to the management and mitigation of various acute and chronic pathologies [18]. Currently, natural products constitute a significant proportion of modern pharmacotherapeutic agents, particularly in the treatment of complex diseases [19]. Historically, they have served as an abundant reservoir of bioactive compounds with therapeutic potential [20]. The advent of high‐throughput screening technologies has substantially accelerated the evaluation of the pharmacological activities of herbal medicines, enhancing their utility in drug discovery pipelines [21]. A promising strategy for elucidating the mechanisms of action of phytochemicals involves the computational prediction of gene regulatory networks modulated by bioactive constituents of medicinal plants [22]. The field of drug discovery is currently facing a pronounced efficacy bottleneck, largely attributable to reductionist paradigms that emphasize single‐target, symptom‐oriented therapies over systems‐level, mechanism‐based interventions [23].

Network pharmacology is rapidly emerging as a transformative paradigm in drug discovery and development, as it synergistically integrates principles of systems biology, bioinformatics, and pharmacology [24]. This integrative in silico approach enables the construction of complex “protein–compound/disease–gene” interaction networks, thereby facilitating the elucidation of the molecular mechanisms underlying the multi‐target and synergistic effects of traditional medicines [18]. Such advancements have catalyzed a shift from the conventional “one drug–one target” paradigm toward a more holistic “network‐target, multi‐component therapeutics” model, better suited for addressing the intricacies of multifactorial diseases [25].

Dengue fever is a rapidly spreading viral infection caused by the dengue virus (DENV), transmitted primarily through the bite of infected *Aedes aegypti* or *Aedes albopictus* mosquitoes. Currently, there is no specific antiviral therapy available for dengue fever; clinical management primarily relies on symptomatic relief, supportive care, and preventive measures. The first licensed vaccine, Dengvaxia, has shown limited efficacy and is associated with a relatively high incidence of adverse effects, which restrict its widespread use. However, it has been noticed that plant‐based herbal drugs have shown great efficacy in treating dengue fever, especially headache, joint, and muscle pain and also the occurrence of severe dengue fever.

*Eucalyptus globulus* has long been used as a mosquito repellent, mainly due to the presence of certain important bioactive compounds such as Eucalyptol. This study was mainly carried out to check the metabolic profile, larvicidal potency of *E.globulus* essential oil against *Aedes aegypti,* and in silico analysis of its metabolites against. Results showed a time‐ and dose‐dependent response of *E. globulus* essential oil against *A.aegepti* larvae, as 90% mortality was noted at 1000 ppm concentration and 120 h. This toxic effect may be attributed to bioactive compounds such as Eucalyptol, Limonene and globulol, which mainly target the GABA receptors in larvae, leading to nervous system breakdown. Eucalyptus essential oils exert their insecticidal action in part by targeting octopaminergic receptors, which are invertebrate‐specific neuroreceptors analogous to adrenergic receptors in vertebrates. These receptors regulate critical physiological processes such as locomotion, metabolism, and nervous system signaling in insects. Disruption of octopaminergic pathways by bioactive components like 1,8‐cineole (Eucalyptol) leads to neural dysfunction, paralysis, and ultimately death in mosquito larvae [26].

In vitro analysis was further confirmed by network pharmacology and molecular docking analysis. Swiss target prediction showed that majority of the drug targets of *E. globulus* essential oil were related to dengue fever, as a total of 70 common targets were revealed by Venn diagrams. These common targets show the potential of metabolites to targets variety of dengue‐related enzymes, signal proteins, and other receptors in humans, which can ultimately lead to the ease of dengue fever‐related symptoms. Further gene ontology analysis of these common targets revealed that highest enrichment of these metabolites was in response to chemical in the biological processes (BP) class. The enrichment of “response to chemical” as the most abundant biological process suggests that dengue‐related targets may be highly responsive to chemical stimuli, including phytochemicals. This highlights the potential of bioactive compounds—such as those in *E. globulus* oil—to modulate key molecular pathways involved in dengue virus transmission or vector survival. Similarly, the prominence of heterocyclic compound binding in the molecular function category indicates a strong interaction potential between target proteins and bioactive molecules with heterocyclic structures. This is particularly relevant to dengue research as many antiviral and insecticidal compounds—including constituents of *E. globulus* oil—possess heterocyclic moieties that may interfere with viral replication or vector physiology. Similarly, metascape analysis of all common disease‐metabolite targets revealed that a significant number of targets were enriched with those related to dengue fever, which makes all the metabolites competitive candidates to be used as drugs against dengue fever (Figure 9).

**FIGURE 9:**
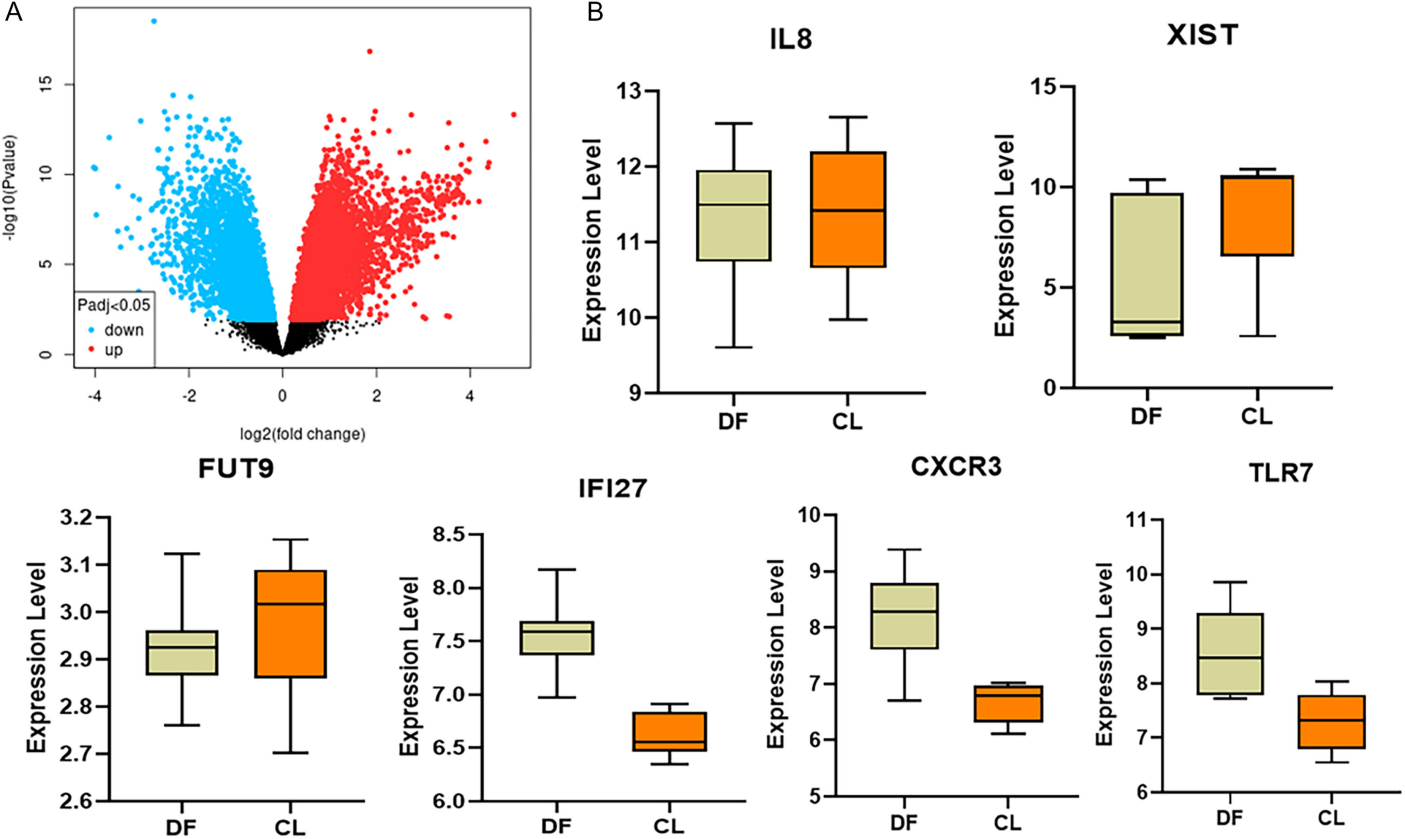
Differential gene expression analysis of Dengue fever (DF) Genes mRNA expression versus control (CL) based on GEO dataset.

Molecular docking analysis shows the binding potential of the selected metabolites with DENV‐2 receptors. The envelope (E) glycoprotein of the dengue virus is essential for mediating viral entry into host cells, facilitating attachment, membrane fusion, and subsequent replication. It also serves as a primary target for neutralizing antibodies, making it a critical component in host immune recognition and antiviral strategies. Targeting the initial stages of dengue virus (DENV) infection, specifically viral attachment and membrane fusion, represents a promising antiviral strategy. These processes are primarily facilitated by the envelope (E) glycoprotein, a class II viral fusion protein that plays a pivotal role in mediating host cell entry [27]. In our results, it was found that all of the metabolites shown binding affinity for DENV‐E glycoprotein (RCSB PDB ID‐1tg8) with the highest binding affinity of Ecalyptol of −7.6 k cal/mol, followed by Globulol −5.9.

## 5 Conclusion

The increasing global burden of dengue virus infections underscores the urgent need for effective antiviral agents. In this study, a comprehensive in silico analysis identified 14 major bioactive compounds from *Eucalyptus globulus* essential oil with potential therapeutic relevance to dengue. Network pharmacology and pathway enrichment analyses revealed significant associations, particularly with DENV‐2 targets. Molecular docking indicated that Globulol and Eucalyptol exhibit strong binding affinities toward key viral proteins (1OKE and 1TG8), suggesting potential inhibitory activity. Moreover, toxicity and drug‐likeness assessments affirmed the safety of these metabolites, supporting their candidacy for further drug development. Overall, the findings highlight the promise of *E. globulus* essential oil as a natural source of anti‐dengue agents, warranting further validation through in vivo studies using appropriate animal models.

## Author Contributions

A.A.: Formal Analysis, Writing original draft, Experimentation. I.A.: Formal Analysis, Writing original draft, Experimentation. S.Q.: Writing: review and editing, Formal analysis, Resources. T.Y.: Writing review and editing, resources, Software. F.U.: Writing: review and editing, Analysis. D.K.: Formal Analysis, Review. Z.U.M.: Supervision, conceptualization. M.: Software, Data analysis, visualization, Writing, Review and Editing. M.H.H.: Supervision, Conceptualization, Writing, Review and editing.

## Funding

The authors have nothing to report.

## Ethics Statement

This study does not involve animals or humans, no ethical approval required. The study involved insect larvae, as the experiments did not involve vertebrate animals, no institutional approval for vertebrate animal experimentation was required. The larvicidal assays were conducted in accordance with standard laboratory procedures and applicable institutional guidelines.

## Consent

The authors have nothing to report.

## Conflicts of Interest

Authors declares no competing academic or financial interests.

## Data Availability

All the data will be made available on a reasonable request.

## References

[1] M. Sallam , H. Eid , N. Awamleh , et al., “Conspiratorial Attitude of the General Public in Jordan Towards Emerging Virus Infections: A Cross‐Sectional Study Amid the 2022 monkeypox Outbreak,” Tropical Medicine and Infectious Disease 7, no. 12 (2022): 411. 10.3390/tropicalmed7120411 PMC9787389 36548666

[2] M. Sallam , D. Dababseh , A. Yaseen , A. Al‐Haidar , N. A. Ababneh , et al., “Conspiracy Beliefs Are Associated With Lower Knowledge and Higher Anxiety Levels Regarding COVID‐19 Among Students at the University of Jordan,” International Journal of Environmental Research and Public Health 17 (2020): 4915. 10.3390/ijerph17144915 PMC7399915 32650409

[3] M. Z. Yousaf , A. Siddique , U. A. Ashfaq , and M. Ali , “Scenario of Dengue Infection & Its Control in Pakistan: An Up—Date and Way Forward,” Asian Pacific Journal of Tropical Medicine 11, no. 1 (2018): 15–23.

[4] R. R. de Almeida , B. Paim , S. A. de Oliveira , et al., “Dengue Hemorrhagic Fever: A State‐of‐the‐Art Review Focused in Pulmonary Involvement,” Lung 195, no. 4 (2017): 389–395. 10.1007/s00408-017-0021-6 PMC7102422 28612239

[5] S. Bhatt , P. W. Gething , O. J. Brady , et al., “The Global Distribution and Burden of Dengue,” Nature 496, no. 7446 (2013): 504–507. 10.1038/nature12060 PMC3651993 23563266

[6] T. M. Conceicão , A. T. Da Poian , and M. H. Sorgine , “A Real‐Time PCR Procedure for Detection of Dengue Virus Serotypes 1, 2, and 3, and Their Quantitation in Clinical and Laboratory Samples,” Journal of Virological Methods 163, no. 1 (2010): 1–9. 10.1016/j.jviromet.2009.10.001 19822173

[7] A. Slonchak , R. Parry , B. Pullinger , et al., “Structural Analysis of 3′UTRs in Insect Flaviviruses Reveals Novel Determinants of sfRNA Biogenesis and Provides New Insights Into Flavivirus Evolution,” Nature Communications 13, no. 1 (2022): 1279. 10.1038/s41467-022-28977-3 PMC8917146 35277507

[8] W. H. Organization , Dengue and Severe Dengue (World Health Organization, 2015, Fact sheet No 117 [updated May 2015]http://www.who.int/mediacentre/factsheets/fs117/en.

[9] O. J. Brady , P. W. Gething , S. Bhatt , J. P. Messina , J. S. Brownstein , et al., “Refining the Global Spatial Limits of Dengue Virus Transmission by Evidence‐Based Consensus,” PLoS Neglected Tropical Diseases, 6, no. 8 (2012). 10.1371/journal.pntd.0001760 PMC3413714 22880140

[10] N. Chandorkar , S. Tambe , P. Amin , and C. Madankar , “A Systematic and Comprehensive Review on Current Understanding of the Pharmacological Actions, Molecular Mechanisms, and Clinical Implications of the Genus Eucalyptus,” Phytomedicine Plus 1, no. 4 (2021): 100089.

[11] N. Immaroh , D. Kuliahsari , and S. Nugraheni , Eucalyptus Globulus Essential Oil Extraction Method (IOP Publishing, 2021), 012103.

[12] M. Yang , J. Chen , L. Xu , et al., “A Network Pharmacology Approach to Uncover the Molecular Mechanisms of Herbal Formula Ban‐Xia‐Xie‐Xin‐Tang,” Evidence‐Based Complementary and Alternative Medicine 2018, no. 1 (2018): 4050714. 10.1155/2018/4050714 PMC6206573 30410554

[13] W. Li , G. Yuan , Y. Pan , C. Wang , and H. Chen , “Network Pharmacology Studies on the Bioactive Compounds and Action Mechanisms of Natural Products for the Treatment of Diabetes Mellitus: A Review,” Frontiers in Pharmacology 08 (2017): 74. 10.3389/fphar.2017.00074 PMC5322182 28280467

[14] Y. Wang , T. Li , and N. Liu , “Mosquito Larval Bioassays,” Cold Spring Harbor Protocols 2023, no. 7 (2023): pdb.prot108040– 10.1101/pdb.prot108040 36882293

[15] M. Wiederstein and M. J. Sippl , “ProSA‐Web: Interactive Web Service for the Recognition of Errors in Three‐Dimensional Structures of Proteins,” Nucleic Acids Research 35, no. Suppl 2 (2007): W407–W410. 10.1093/nar/gkm290 PMC1933241 17517781

[16] N. Xiong and Q. Sun , “Identification of Stage‐Related and Severity‐Related Biomarkers and Exploration of Immune Landscape for Dengue by Comprehensive Analyses,” Virology Journal 19, no. 1 (2022): 130. 10.1186/s12985-022-01853-8 PMC9344228 35918744

[17] B. Zhou , J. F. Xiao , L. Tuli , and H. W. Ressom , “LC‐MS‐Based Metabolomics,” Molecular BioSystems 8, no. 2 (2012): 470–481. 10.1039/c1mb05350g PMC3699692 22041788

[18] Gaurav , S. Zahiruddin , B. Parveen , et al., “TLC‐MS Bioautography‐Based Identification of Free‐Radical Scavenging, α‐Amylase, and α‐‍Glucosidase Inhibitor Compounds of Antidiabetic Tablet BGR‐34,” ACS Omega 5, no. 46 (2020): 29688–29697. 10.1021/acsomega.0c02995 PMC7689676 33251404

[19] S. K. Pal and Y. Shukla , “Herbal Medicine: Current Status and the Future,” Asian Pacific Journal of Cancer Prevention 4, no. 4 (2003): 281–288. 14728584

[20] A. Rehman , U. A. Ashfaq , M. R. Javed , F. Shahid , F. Noor , and S. Aslam , “The Screening of Phytochemicals Against NS5 Polymerase to Treat Zika Virus Infection: Integrated Computational Based Approach,” Combinatorial Chemistry & High Throughput Screening 25, no. 4 (2022): 738–751. 10.2174/1386207324666210712091920 34254908

[21] N. Tan , K. A. Gwee , J. Tack , et al., “Herbal Medicine in the Treatment of Functional Gastrointestinal Disorders: A Systematic Review With Meta‐Analysis,” Journal of Gastroenterology and Hepatology 35, no. 4 (2020): 544–556. 10.1111/jgh.14905 31674057

[22] L. Shao and B. Zhang , “Traditional Chinese Medicine Network Pharmacology: Theory, Methodology and Application,” Chinese Journal of Natural Medicines 11, no. 2 (2013): 110–120. 10.1016/S1875-5364(13)60037-0 23787177

[23] A. I. Casas , A. A. Hassan , S. J. Larsen , et al., “From Single Drug Targets to Synergistic Network Pharmacology in Ischemic Stroke,” Proceedings of the National Academy of Sciences 116, no. 14 (2019): 7129–7136. 10.1073/pnas.1820799116 PMC6452748 30894481

[24] Y. Dong , L. Hao , K. Fang , Han X‐x , H. Yu , et al., A Network Pharmacology Perspective for Deciphering Potential Mechanisms of Action of *Solanum nigrum L.* in Bladder Cancer, BMC Complementary Medicine and Therapies 21, no. 1 (2021): 1–14. 10.1186/s12906-021-03215-3 PMC7836472 33494738

[25] U. Chandran , N. Mehendale , S. Patil , R. Chaguturu , and B. Patwardhan , “Network Pharmacology,” Innovative Approaches in Drug Discovery (2016): 127.

[26] A. V. Costa , P. D. F. Pinheiro , V. T. de Queiroz , et al., “Chemical Composition of Essential Oil From *Eucalyptus* *c* *itriodora* Leaves and Insecticidal Activity Against *Myzus* *p* *ersicae* and *Frankliniella* *s* *chultzei* ,” Journal of Essential Oil Bearing Plants 18, no. 2 (2015): 374–381.

[27] Y. Modis , “Class II Fusion Proteins,” Viral Entry into Host Cells (2013): 150–166. 10.1007/978-1-4614-7651-1_8 PMC7123093 23884590

